# Independently Evolving Species in Asexual Bdelloid Rotifers

**DOI:** 10.1371/journal.pbio.0050087

**Published:** 2007-03-20

**Authors:** Diego Fontaneto, Elisabeth A Herniou, Chiara Boschetti, Manuela Caprioli, Giulio Melone, Claudia Ricci, Timothy G Barraclough

**Affiliations:** 1 Dipartimento di Biologia, Università di Milano, Milan, Italy; 2 Division of Biology, Imperial College London, Ascot, United Kingdom; 3 Natural Environment Research Council Centre for Population Biology, Imperial College London, Ascot, United Kingdom; 4 Institute of Biotechnology, University of Cambridge, Cambridge, United Kingdom; 5 Jodrell Laboratory, Royal Botanic Gardens, Kew, United Kingdom; Duke University, United States of America

## Abstract

Asexuals are an important test case for theories of why species exist. If asexual clades displayed the same pattern of discrete variation as sexual clades, this would challenge the traditional view that sex is necessary for diversification into species. However, critical evidence has been lacking: all putative examples have involved organisms with recent or ongoing histories of recombination and have relied on visual interpretation of patterns of genetic and phenotypic variation rather than on formal tests of alternative evolutionary scenarios. Here we show that a classic asexual clade, the bdelloid rotifers, has diversified into distinct evolutionary species. Intensive sampling of the genus *Rotaria* reveals the presence of well-separated genetic clusters indicative of independent evolution. Moreover, combined genetic and morphological analyses reveal divergent selection in feeding morphology, indicative of niche divergence. Some of the morphologically coherent groups experiencing divergent selection contain several genetic clusters, in common with findings of cryptic species in sexual organisms. Our results show that the main causes of speciation in sexual organisms, population isolation and divergent selection, have the same qualitative effects in an asexual clade. The study also demonstrates how combined molecular and morphological analyses can shed new light on the evolutionary nature of species.

## Author Summary

The evolution of distinct species has often been considered a property solely of sexually reproducing organisms. In fact, however, there is little evidence as to whether asexual groups do or do not diversify into species. We show that a famous group of asexual animals, the bdelloid rotifers, has diversified into distinct species broadly equivalent to those found in sexual groups. We surveyed diversity within a single clade, the genus *Rotaria,* from a range of habitats worldwide, using DNA sequences and measurements of jaw morphology from scanning electron microscopy. New statistical methods for the combined analysis of morphology and DNA sequence data confirmed two fundamental properties of species, namely, independent evolution and ecological divergence by natural selection. The two properties did not always coincide to define unambiguous species groups, but this finding is common in sexual groups as well. The results show that sex is not a necessary condition for speciation. The methods offer the potential for increasing our understanding of the nature of species boundaries across a wide range of organisms.

## Abstract 3

New analyses of genetic and morphological data reveal that the bdelloid rotifers, a famous asexual group, have diversified into distinct species and that sex is not a necessary condition for speciation.

## Introduction

Species are fundamental units of biology, but there remains uncertainty on both the pattern and processes of species existence. Are species real evolutionary entities or convenient figments of taxonomists' imagination [1–3]? If they exist, what are the main processes causing organisms to diversify [1,4]? Despite considerable debate, surprisingly few studies have formally tested the evolutionary status of species [1,5,6].

One central question concerning the nature of species has been whether asexual organisms diversify into species [1]. The traditional view is that species in sexual clades arise mainly because interbreeding maintains cohesion within species, whereas reproductive isolation causes divergence between species [7]. If so, asexuals might not diversify into distinct species, because there is no interbreeding to maintain cohesive units above the level of the individual. However, if other processes were more important for maintaining cohesion and causing divergence, for example, specialization into distinct niches, then asexuals should diversify in a manner similar to sexuals, although the rate and magnitude of divergence might differ [8–11].

Empirical evidence to test these ideas has been rare. Most asexual animal and plant lineages are of recent origin [9,12]. The diffuse patterns of variation typical of such taxa [13] could simply reflect their failure to survive long enough for speciation to occur or the effects of ongoing gene flow from their sexual ancestors [9,12]. Distinct genetic and phenotypic clusters have been demonstrated in bacteria [14–17] and discussed as possible evidence for clonal speciation [1]. However, all the study clades engage in rare or even frequent recombination as well as clonal reproduction [14,18,19]. Although horizontal gene transfer can occur between distantly related bacteria, homologous recombination occurs only at appreciable frequency between closely related strains [20,21]. Therefore, clusters in these bacteria could arise from similar processes to interbreeding and reproductive isolation in sexual eukaryotes [20]. Aside from issues of sexuality, previous studies looking for distinct clusters have been descriptive, relying on visual interpretation of plots of genetic or phenotypic variation rather than on formal tests of predictions under null and alternative evolutionary scenarios [1].

Here, we demonstrate that a classic asexual clade, the bdelloid rotifers, has diversified into independently evolving and distinct entities arguably equivalent to species. Bdelloids are abundant animals in aquatic or occasionally wet terrestrial habitats and represent one of the best-supported clades of ancient asexuals [22–24]. They reproduce solely via parthenogenetic eggs, and no males or traces of meiosis have ever been observed. Molecular evidence that bdelloid genomes contain only divergent copies of nuclear genes present as two similar copies (alleles) in diploid sexual organisms rules out anything but extremely rare recombination [25–27]. Yet, bdelloids have survived for more than 100 million y and comprise more than 380 morphologically recognizable species and 20 genera [28]. The diversity of the strictly asexual bdelloids poses a challenge to the idea that sex is essential for long-term survival and diversification [29]. However, taxonomy does not constitute strong evidence for evolutionary species: the species could simply be arbitrary labels summarizing morphological variation among a swarm of clones [7]. We adopt a general evolutionary species concept, namely, that species are independently evolving and distinct entities, and then break the species problem into a series of testable hypotheses derived from population genetic predictions [3]. We use the word “entity” to refer to a set of individuals comprising a unit of diversity according to a given criterion or test: the question of whether to call those entities “species” will be returned to below.

**Figure 1:**
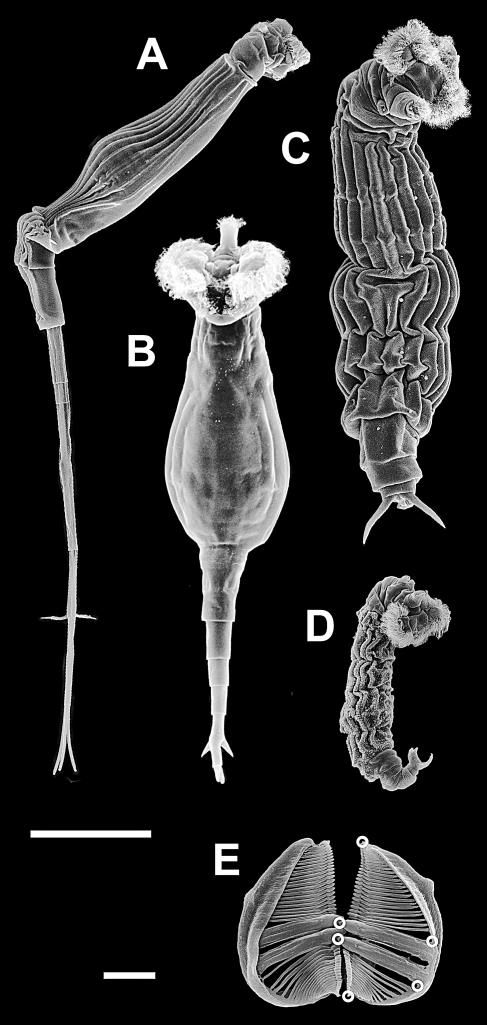
SEM Pictures of Some Species of the Genus *Rotaria* (A) R. neptunia, lateral view; (B) R. macrura, ventral view; (C) R. tardigrada, dorsal view; (D) R. sordida, lateral view; and (E) trophi of R. tardigrada with open circles showing the location of landmarks used for the shape analysis. Scale bars: 100 μm for animals, 10 μm for trophi.

**Figure 2:**
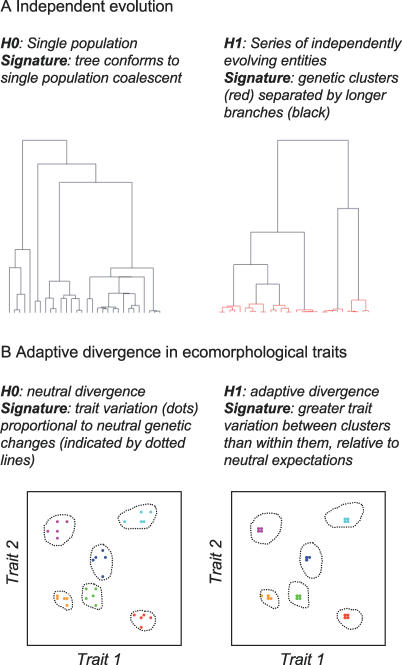
Scheme Showing the Predicted Patterns of Genetic and Morphological Variation Underlying Our Tests of Alternative Scenarios of Diversification (A) Hypothetical trees showing expected genetic relationships among a sample of individuals under the null model that the sample is drawn from a single asexual population (H0) and under the alternative model that the clade has diversified into a set of independently evolving entities (H1). (B) Expected variation in two ecomorphological traits evolving either neutrally (H0) or by adaptive divergence (H1) in a genus that has diversified into six genetic clusters. Note that a mixed pattern is possible: Some genetic clusters may have experienced divergent selection on morphology, whereas others have not.

Focusing on the genus *Rotaria* (Figure 1), one of the best-characterized genera of bdelloids, we use combined molecular and morphological analyses to distinguish alternative scenarios for bdelloid diversification (Figure 2). First, the entire clade might represent a single species, that is, a swarm of clones with no diversification into independently evolving subsets of individuals. Second, the clade may have diversified into a series of independently evolving entities. By “independently evolving,” we mean that the evolutionary processes of selection and drift operate separately in different entities [8,9], such that genotypes can only spread within a single entity. Possible causes of independence include geographical isolation or adaptation to different ecological niches [10,17]. The expected outcome is cohesion within entities but genetic and phenotypic divergence between them [9–11].

We first test for the presence of independently evolving entities. Under the null scenario of no diversification, genetic relationships should conform to those expected for a sample of individuals from a single asexual population (H0, Figure 2A). Under the alternative scenario that independently evolving entities are present, we expect to observe distinct clusters of closely related individuals separated by long branches from other such clusters (H1, Figure 2A; and [9]). Coalescent models can be used to distinguish the two scenarios [30]. Failure to reject the null model would indicate a lack of evidence for the existence of independently evolving entities.

Next, to investigate the role of adaptation to different niches in generating and maintaining diversity within the clade, we extend classic methods from population genetics to test directly for adaptive divergence of ecomorphological traits. If trait diversity evolves solely by neutral divergence in geographic isolation, we expect morphological variation within and between entities to be proportional to levels of neutral genetic variation (H0, Figure 2B, Materials and Methods). If, instead, different entities experience divergent selection on their morphology, we expect greater morphological variation between clusters than within them, relative to neutral expectations (H1, Figure 2B; and [31]). Past work has often discussed sympatry of clusters as evidence for niche divergence [1], but, in theory, coexistence can occur without niche differences [32]; hence, we introduce an alternative, more direct approach.

Our results demonstrate that bdelloids have diversified not only into distinct genetic clusters, indicative of independent evolution, but also into entities experiencing divergent selection on feeding morphology, indicative of niche divergence. In common with findings of cryptic species in sexual organisms [33,34], the morphologically coherent groups experiencing divergent selection often include several genetic clusters: this introduces difficulties in deciding which units to call species, but this problem is shared with sexual organisms [3,33]. In short, bdelloids have diversified into entities equivalent to sexual species in all respects except that individuals do not interbreed. The results demonstrate the benefits of statistical analyses of combined molecular and morphological data for exploring the evolutionary nature of species.

## Results/Discussion

**Figure 3:**
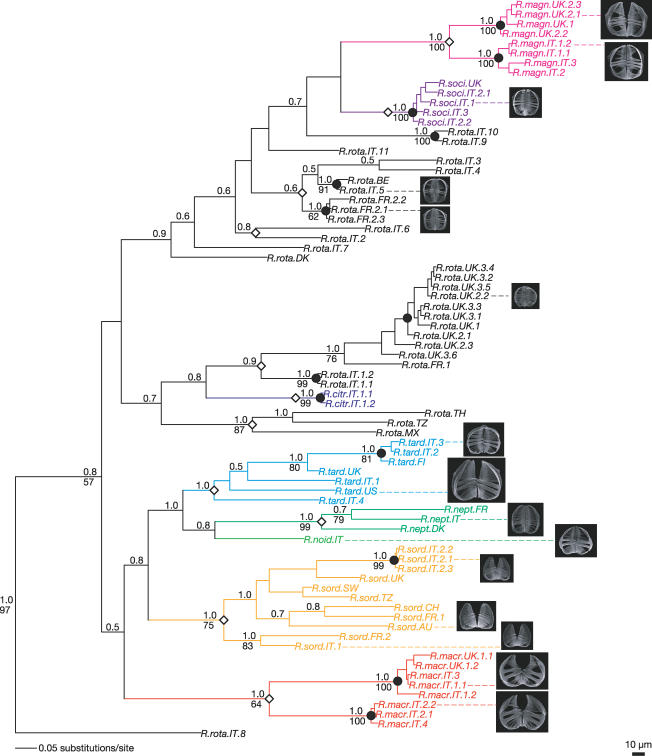
Phylogenetic Relationships in the Genus *Rotaria* The consensus of 80,000 sampled trees from Bayesian analysis of the combined *cox1* and *28S* rDNA data sets is shown, displaying all compatible groupings and with average branch lengths proportional to numbers of substitutions per site under a separate GTR + invgamma substitution model for the *cox1* and *28S* partitions. Posterior probabilities above 0.5 and bootstrap support above 50% from a maximum parsimony bootstrap analysis are shown above and below each branch, respectively. Support values for within-species relationships are not shown for very short branches but are shown in Figures S1 through S3. Closed circles indicate clusters identified by the clustering analysis. Colors represent traditional species memberships. Diamonds indicate taxonomic species and monophyletic groups of *Rotaria*. Names refer to the species, the country, the number of site within that country for that species, and the number of individual from that site if several were isolated; for example, R.macr.IT.1.1 refers to the first individual from site 1 in Italy for R. macrura. Pictures of trophi from one individual from each cluster are shown to scale: Representatives of all sampled populations are shown in Figure S4. A full list of names and localities of samples is available in Table S1.

We collected all individuals of *Rotaria* encountered during 3 y searching rivers, standing water, dry mosses, and lichens, centered on Italy and the United Kingdom but also globally [35]. Individuals were identified to belong to nine taxonomic species (Tables S1 and S2). Most of the described species of *Rotaria* missing from our sample are known from only one record or are very rarely encountered (Protocol S1). Bayesian and maximum parsimony analyses of mitochondrial cytochrome oxidase I *(cox1)* and nuclear 28S ribosomal DNA sequences provide strong support for the monophyly of taxonomic species (Figures 3, S1, S2, and S3 and Text S1), with the sole exception of *R. rotatoria,* which was already suspected to comprise a species complex based on disagreements among authors [36,37].

**Figure 4:**
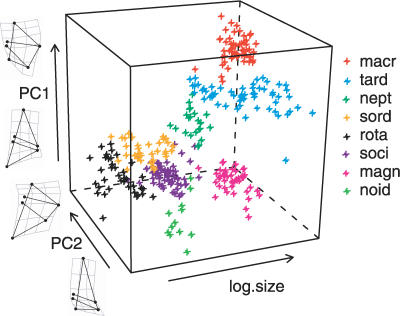
Plot of the Size and Shape (the First Two PCs, PC1 and PC2, from the Generalized Procrustes Analysis) of Trophi across Species The directions of shape variation along each axis are shown for PC1 and PC2, respectively, using ×2 and ×4 magnification of the observed variation for emphasis. PC1 represents a continuum from oval to rounder trophi and from parallel to converging major teeth. PC2 represents a trend in the distance of the major teeth from the attachment point between the two halves of the trophi.

Morphometric analyses further support the distinctness of taxonomic species. Bdelloid morphology is hard to measure because of their shape-changing abilities; hence, we used geometric morphometrics [38] to measure the only suitable trait, their hard jaws, called trophi [39] (Figures 1 and S4). Trophi size and shape are not characters that have been used in the traditional taxonomy of the genus (Table S2). Trophi scale weakly with rough measures of body size of each species (mean trophi size against log body length from [37]: *r* = 0.55, *p* = 0.2, Spearman's rank test), and both the size and shape of trophi likely reflect different types or sizes of particulate food consumed, although the details of how food is processed remain unclear [28]. Discriminant analysis of the first five principal components (PCs) describing trophi shape (cumulative explained variance, 97.1%; Materials and Methods) produced a correct classification with respect to traditional taxonomy of most specimens of *R. macrura, R. neptunia, R. sordida,* and R. tardigrada (Table S3). The remaining species overlapped in shape but could be discriminated by size (Figures 4 and S5). Related species on the DNA trees tend to have similar morphology: for example, *R. magnacalcarata, R. socialis,* and *R. rotatoria FR.2.1* and *IT.5* overlap in shape, but are more distant from *R. rotatoria UK.2.2*. Only two of the traditional species found to be monophyletic in the DNA tree displayed significant variation in size or shape among populations: R. sordida and R. tardigrada. In both cases, the populations that differed were deeply divergent in the DNA tree as well.

Congruence between molecules and morphology confirms that most traditional *Rotaria* species are monophyletic clades but does not rule out the possibility that taxa reflect variation within a single asexual species or swarm of evolutionarily interacting clones. Under the alternative scenario that independently evolving entities are present, we expect to observe clusters of closely related individuals separated from other such clusters by longer internal branches on a DNA tree [9,30,40]. We therefore tested for significant clustering by comparing two models describing the likelihood of the branching pattern of the DNA trees: first, a null model that the entire sample derives from a single population following a neutral coalescent [41], and, second, a model assuming a set of independently evolving populations joined by branching that reflects the timing of divergence events between them, that is, cladogenesis [9,30,42]. The models allow departures from strict assumptions of constant population size and rates of cladogenesis (see Materials and Methods).

The results indicate significant clustering within *Rotaria,* as expected if several independently evolving entities are present and consistent with patterns of mtDNA diversity from a broad sample of bdelloids [43]. The maximum likelihood solution for the independent evolution model on the combined tree infers 13 isolated clusters, with the remaining individuals inferred to be singletons (Figure 3; Table S4). Two monophyletic taxonomic species contained two separate clusters: R. magnacalcarata has two clusters corresponding to the U.K. and Italian samples, whereas R. macrura has two clusters not matching sampling locality. Uncorrected pairwise distances of *cox1* within clusters ranged from 0% to 3.3% (mean, 1.5%), and those between clusters ranged from 4.1% to 23.1% (mean, 16.0%). The null model that the entire lineage represents a single cluster can be rejected (log likelihood ratio test, 2 × ratio = 30.8, χ^2^ test, three degrees of freedom, *p* < 0.0001).

Our results indicate that independently evolving entities are present in bdelloids but at a lower level than taxonomic species, that is, cryptic taxa within the taxonomic species. However, the nature of independent evolution remains unclear. Clusters might simply represent geographically isolated, or even partially geographically isolated, populations evolving neutrally [32,44]. Alternatively, the clade might have diversified into ecologically distinct species experiencing divergent selection pressures. To resolve these alternatives, we test directly for divergent selection between different lineages, adapting classic methods from molecular population genetics [31,45]. If rotifers have experienced divergent selection on trophi morphology between species, for example, adapting to changes in habitat or resource use, we expect low variation within species and high variation between species, relative to the same ratio for neutral changes.

**Figure 5:**
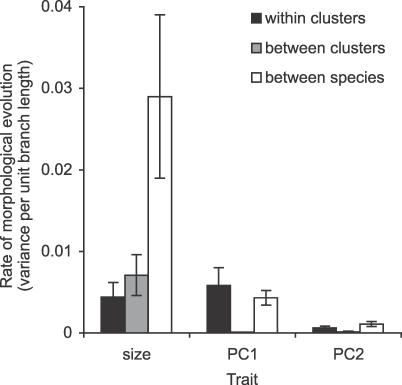
Evolutionary Rates of Changes in Trophi Size and Shape Rates are expressed as the variance in each trait per unit branch length. Branch lengths are in units of the number of silent substitution per codon of *cox1*. Estimates from the maximum model with three rate classes are shown: within clusters, between clusters within taxonomic species, and between taxonomic species. Error bars show confidence limits within 2 log likelihood units of the maximum likelihood solution. Hierarchical likelihood ratio tests indicated that the model for size could be simplified to assume a joint rate for within cluster and between cluster branches (Table S5).

To explore the level at which divergent selection acts on morphology, we compared rates of morphological change within clusters, between clusters within taxonomic species, and between taxonomic species, in each case relative to silent substitution rates in *cox1,* assumed to reflect neutral changes (see Materials and Methods). The test is robust to sampling issues and differences in mutational mechanism between morphology and *cox1* (see Materials and Methods). The results reveal significant evidence for divergent selection on trophi size and PC2 (Figure 5; Table S5). However, divergent selection occurs between taxonomic species, not between clusters; both traits are conserved within taxonomic species but diverge rapidly between species, relative to neutral expectations. Changes in PC1 are more complex, being lower between clusters either than within clusters or between taxonomic species. However, overall the results demonstrate divergent selection on the size and some aspects of shape of the trophi.

Our results show that *Rotaria* has undergone adaptive diversification in feeding morphology, presumably associated with specialization to different habitats. The finding is supported by observations of ecological differences among the traditional species. For example, R. socialis and R. magnacalcarata live externally on the body of the water louse Asellus aquaticus but partition their use of the host, with the former living around the leg bases and the latter on the anterior, ventral surface. Our analyses show that these traditional species, which are found living together on single louse individuals, are evolutionarily independent and distinct entities. Another traditional species, *R. sordida,* is found in more terrestrial habitats than the other species, although it sometimes co-occurs with *R. tardigrada,* which is generally more aquatic (Table S2). Therefore, informal observations of habitat partitioning and coexistence at local scales add further support to the role of niche partitioning.

Not all of the entities identified as genetic clusters display evidence of divergent selection on feeding morphology: the signature of divergent selection was detected at a broader level than that of independently evolving clusters. One possible explanation is that some clusters arose solely from neutral divergence in complete or partial geographical isolation [32,44]. Some of the clusters do comprise geographically localized sets of samples, but at least one traditional species, *R. macrura,* contains two clusters without obvious geographical separation. Alternatively, divergent selection might act at different hierarchical levels on different traits [17]: clusters might have diverged in unmeasured traits such as behavior, gross body morphology, or life history. Future work sampling additional genetic markers and phenotypic traits for the identified clusters might distinguish these alternatives.

So which level should we call “species”? As increasingly recognized in reviews of species concepts, the answer will depend on which aspect of diversity is of most interest and on the intended use of the delimitation [3,46]. For evolutionary studies, for example, into how bdelloids might adapt to changing environments, the genetic clusters provide statistical evidence of independent evolution within the traditionally recognized species that needs to be taken into account. For ecological studies, the traditional species conform closely to units that are ecologically distinct in terms of feeding morphology. Perhaps surprisingly for a poorly studied group of microscopic animals, traditional species limits appear to be robust for many purposes with the exception of the paraphyletic R. rotatoria. However, the important point here is that the same issues apply to studies of sexual organisms. Genetic surveys often reveal cryptic species within morphologically coherent sexual species and elicit the same arguments over their interpretation [3,33,34].

We conclude that bdelloids display the same qualitative pattern of genetic and morphological clusters, indicative of diversification into independently evolving and distinct entities, as found in sexual clades. This refutes the idea that sex is necessary for diversification into evolutionary species. Similar approaches could be used to explore the nature of species in sexual clades—for example, how often is speciation accompanied by ecological divergence compared to a null model of reproductive isolation and neutral divergence [32,47,48]? In addition, clades differing in levels of recombination could be compared to determine how sexual reproduction affects the strength and rate of diversification. Does the requirement for reproductive isolation limit opportunities for speciation in sexuals, or do their faster adaptive rates promote stronger patterns of diversification than in asexuals [9,49]? Microbial eukaryotes, prokaryotes, and fungi could provide additional study clades for such studies [12], linked to genetic studies verifying the presumed lack of recombination [50].

Our study highlights the advantages of statistical analyses of combined morphological and molecular data. Recent work delimiting or identifying species from DNA barcode data [34,51] has been criticized for relying on organelle genome markers, which may not reveal recent divergences or reflect the history of nuclear genes [52,53]. Morphology provides a ready window on adaptive differences between populations, often the first sign of divergence and at the present easier to sample than the genes underlying important traits [54,55], but has lacked the theoretical framework of DNA. Combined analyses, sampling at the population level across entire clades, offer new potential to uncover the nature of species and biological diversity. Our methods could be readily applied to sexual clades and to other cases presenting challenges to current theories, such as groups in which barriers to interbreeding appear to be weak or nonexistent [1].

## Materials and Methods

### DNA analyses.

DNA was isolated either from clonal samples of five to 25 individuals grown in the laboratory from a single wild-caught individual or from single wild-caught individuals using a chelex preparation (InstaGene Matrix; Bio-Rad, http://www.bio-rad.com). The 28S rDNA and *cox1* mtDNA were amplified and sequenced by PCR as described in Protocol S1. Trees were reconstructed from the *cox1* and *28S* rDNA matrices separately and from a combined matrix for all individuals with at least one gene sequenced. Bayesian analyses were run in Mr Bayes (http://mrbayes.csit.fsu.edu) 3.1.1 for 5 million generations with two parallel searches, using a general transition rate (GTR) + invgamma model [56]. The combined analysis implemented a partition model with a separate GTR + invgamma model and rate parameter for the two partitions. Maximum parsimony support was assessed using 100 bootstrap replicates, searching each heuristically with 100 random addition replicates and TBR branch swapping in Paup*4.10. Eight individuals from the related genus *Dissotrocha* were included as outgroups. Comparisons of the two genes are described in Protocol S1 and Text S1.

### Morphometric analyses.

Trophi were prepared for scanning electron microscopy (SEM) by dissolving soft tissues on a cover slide with sodium hypochloride (NaOCl 4%), rinsing with deionized water, dehydrating at room temperature, and sputter-coating a thin layer of gold. Shape was measured by Generalized Procrustes Analysis (GPA) [57] of six landmarks on digitized pictures of the cephalic (ventral) view (Figure 1). GPA coordinates were used for PC analysis after projection onto an Euclidean space tangent to the shape space (see Protocol S1). Size was expressed as centroid size of the landmark configuration. We attempted to culture all individuals, to allow morphometrics and sequencing on individuals from the same clone. However, not all clones survived in the laboratory; for these, we used replicate individuals from the same wild population where possible. In total, we measured 326 SEM pictures of trophi from 23 populations belonging to eight species (see Table S1b). For species with both laboratory-cultured and wild-caught measures, we found no evidence that sample type influenced either the mean or variance of size and shape measures (Table S6), indicating respectively that species differences are genetically based (not environmental) and that there appears to be little genetic variation for morphology within populations. Statistical analyses were performed using the R statistical programming language [58] and routines in the Tps series of programs [59].

### Clustering test for independent evolution.

Under the null model that the entire sample derives from a single population obeying a single coalescent process, we calculated the likelihood of waiting times, *x_i_,* between successive branching events on the DNA tree as

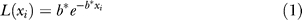
with

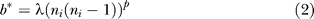
where *n_i_* is the number of lineages in waiting interval *i,* λ is the branching rate for the coalescent (the inverse of twice the effective population size in a neutral coalescent), and *p* is a scaling parameter that allows the apparent rate of branching to increase or decrease through time, fitting a range of qualitative departures from the strict assumptions of a neutral coalescent, for example, growing (*p* < 1) or declining (*p* > 1) population size [30]. Under the alternative model that the sample derives from a set of independently evolving populations, each one evolving similarly to the null case, we calculated the likelihood of waiting times as Equation 6 from Pons et al. [30]. The alternative model optimizes a threshold age, *T,* such that nodes before the threshold are considered to be diversification events with branching rate *λ*_D_ and scaling parameter *p*_D_. Branches crossing the threshold define *k* clusters each obeying a separate coalescent process but with branching rate, *λ*_C_, and scaling parameter, *p*_C_, assumed to be constant across clusters. The alternative model thus has three additional parameters. Models were fitted using an R script available from T.G.B. to an ultrametric tree obtained by rate smoothing the combined analysis DNA tree using penalized likelihood in r8s (http://ginger.ucdavis.edu/r8s) and cross-validation to choose the optimal smoothing parameter for each tree [60].

### Test for divergent selection.

In an asexual clade, all genes have the same underlying genealogy: the entire genome is inherited as a single unit. Assuming that silent substitutions are neutral, the expected number of silent mtDNA substitutions on a branch of the genealogy is *μt,* where *t* is the branch length in units of time and *μ* is the mutation rate of the gene. Assuming a neutral morphological trait evolving by Brownian motion, the expected squared change (variance) along a branch is 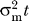 *,* where 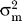 is the mutational rate of increase of variance [61]. The expectations are the same for branches within populations or between them. Therefore, the average rate of change of a neutral trait expressed as variance per silent substitution should be the same within populations as between them, that is, 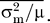 This prediction holds even if mutation rates vary across the tree, providing they do so without a systematic bias between the branch classes being compared, a reasonable assumption shared with widely used molecular versions of the test [31].

We reconstructed evolutionary changes in trophi size and shape (PC1 and PC2) onto the DNA tree using the Brownian motion model by Schluter et al. [62] implemented in the Ape library for R [63]. Branch lengths were optimized as the proportion of silent substitutions per codon using PAML software [64]. The null model assumes a constant rate of morphological change across the entire tree. The alternative model labels branches as between taxonomic species, within species and within clusters, and estimates different rates for each class. Under a three rate-class model, the likelihood of the reconstruction, Equation 3 of [62] becomes the product of the equivalent likelihood for each class of branches.

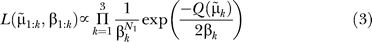
where *k* indicates the branch classes from 1 to 3, *β_k_* is the rate parameter for each class of branches, *N_k_* is the number of nodes ancestral to each class of branch, and *Q*(*ũ_k_*) is the sum of the scaled variance of changes across branches [62] of class *k*. Optimization was implemented in a modified version of the “ace” function of Ape, available from T. G. B. Divergent selection between taxonomic species, for example, would be indicated by a significantly lower rate within cluster and within species branches (classes 1 and 2) than between species branches (class 3). Assumptions and robustness of the test are discussed further in Protocol S2.

## Supporting Information

Figure S1 Phylogenetic Relationships from Bayesian Analysis of the Combined Data Posterior probabilities from the Bayesian analysis are indicated next to each node. Below the branch are bootstrap percentages from a maximum parsimony search with 100 bootstrap replicates each using a heuristic search with 100 random addition replicates, TBR branch swapping, and saving only one tree per addition replicate.
(21 KB PDF)

Figure S2 Phylogenetic Relationships from Bayesian Analysis of *cox1*
Posterior probabilities are indicated above each branch; parsimony bootstrap values are indicated below each branch.
(20 KB PDF)

Figure S3 Phylogenetic Relationships from Bayesian Analysis of 28S rDNA Posterior probabilities are indicated above each branch; parsimony bootstrap values are indicated below each branch.
(14 KB PDF)

Figure S4 SEM Pictures of Trophi from Each Study Population (A) R. macrura macrIT2; (B) R. macrura macrIT1; (C) R. macrura macrIT3; (D) R. macrura R.macr.UK.1; (E) R. magnacalcarata magnIT1; (F) R. magnacalcarata magnIT3; (G) R. magnacalcarata magnIT2; (H) R. magnacalcarata R.magn.UK.2.1; (I) R. socialis sociIT1; (J) R. socialis sociIT2; (K) R. socialis sociIT3; (L) R. socialis R.soci.UK, (M) R. rotatoria R.rota.IT.5; (N) R. rotatoria R.rota.FR.2.1; (O) R. rotatoria R.rota.UK.2.2; (P) R. sordida sordIT1; (Q) R. sordida sordIT2; (R) R. sordida sordAU; (S) R. neptunoida noidIT; (T) R. neptunia R.nept.IT, (U) R. tardigrada tardIT1; (V) R. tardigrada tardIT3; (W) R. tardigrada R.tard.US; and (X) landmarks and links used for shape analysis.
(197 KB PDF)

Figure S5 Box Plot of the Size of Trophi for Each Study Population Analysis of variance test, ln CS: *F*_22,303_ = 684.17, *p* < 0.0001.
(56 KB PDF)

Protocol S1 Sampling, Molecular Analyses, and Morphometrics (87 KB PDF)

Protocol S2 Test for Divergent Selection on Morphology (72 KB PDF)

Table S1 Locality Records for DNA Sequences and Morphometric Measurements (78 KB PDF)

Table S2 Traditional Taxonomy of *Rotaria* Species (60 KB PDF)

Table S3 Discriminant Analysis of Trophi Shape (29 KB PDF)

Table S4 Comparison of Models of Single versus Multiple Independently Evolving Entities (47 KB PDF)

Table S5 Comparison of Alternative Models for Rates of Changes in Trophi Size and Shape within and between Clusters and Species (37 KB PDF)

Table S6 The Effects of Sampling Type (Clonal versus Population Sample) on the Mean and Variance of Size and Shape of Trophi (39 KB PDF)

Text S1 Comparison of *cox1* and 28S rDNA Results (57 KB PDF)

### Accession Numbers

DNA sequences have been deposited at GenBank (http://www.ncbi.nlm.nih.gov/Genbank) under accession numbers DQ656756 to DQ656882.

## Abbreviations

GTR: general transition rate
PC: principal component
SEM: scanning electron microscopy
