# Supplementary material for: Independently Evolving Species in Asexual Bdelloid Rotifers
Source: PLoS Biol. 2007 Mar 20;5(4):e87. doi: 10.1371/journal.pbio.0050087 (PMC1828144; doi:10.1371/journal.pbio.0050087)
Supplement: Supplementary file 1 [file pbio.0050087.sd001.pdf]

PROTOCOL S1

Sampling. Our sample included nine out of twenty-four taxonomically described species of *Rotaria*. Of the species not encountered in our sample, 6 are known from only a single record, 2 from only 2 records, 5 are rarely encountered, 1 was encountered once but culturing and DNA extraction failed and 1 is unreliably distinguished from *R. rotatoria*. We did not bias our sample towards particular species, rather collected every *Rotaria* we found from as wide a range of habitats as possible where we anticipated the genus would occur. We attempted to culture every individual we found, to allow morphological measurements and DNA sequencing on individuals from the same clonal lineage. However, not all clones survived in the laboratory, and for these we used replicate individuals from the same wild population for sequencing and morphometrics. Similarly, it was not possible to obtain all data for all samples hence our three datasets comprise incompletely overlapping sets of individuals (Table S1).

Species identification. Bdelloids can only be reliably identified by long and careful examination of living animals. The relevant characters often contract on preservation and therefore few type specimens of bdelloids are available. Moreover, bdelloid taxonomy has never progressed beyond a subjective approach. Nevertheless, we followed the most recent taxonomic treatment of bdelloids (1) to name every animal we found. Species in the genus *Rotaria* can be characterized by a number of morphological traits recognizable from living animals (2) (Fig.1, Table S2). Most of the species are quite well defined, whereas *Rotaria rotatoria* and *R. sordida* show variability in body traits between different populations and authors do not agree on the taxonomic status of these morphotypes (1, 3).

DNA extraction and sequencing. We isolated DNA using a chelex preparation (InstaGene Matrix, Bio-Rad) as per manufacturers instruction with volumes varying from 15 to 30 µl depending of the number of animals used. Cytochrome oxidase I (*cox1*) was PCR amplified using optimized primers Hcox1 (5'-GGT CAA CAA ATC ATA AAG ATA TTG G-3') and Lcox1 (5'-TAA ACT TCA GGG TGA CCA AAA AAT CA-3'). Cycle conditions comprised initial denaturing at 94ºC for 5 minutes, followed by 35 cycles of 94ºC for 1 minute, 40ºC for 1 minute and 72ºC for 90 seconds, and a final extension step of 72ºC for 5 minutes. The D1-D4 region of 28S rDNA was amplified using the primers D1F (5'-CCC GCT GAA TTT AAG CAT AT-3') and REV (5'-TAG ATG GTT CGA TTA GTC TTT CGC-3'). Nested PCRs where performed where necessary using the primers 28SFOR (5'-AAC AAG TAC CGT GAG GGA AAG TTG-3') and D3R (5'-TAG TTC ACC ATC TTT CGG GTC-3'). Cycle conditions

comprised an initial denaturation step of 95ºC for 5 minutes, followed by 3 cycles of 94ºC for 30 seconds, 45ºC for 20 seconds, 72ºC for 30 seconds, then similar cycles but changing the annealing temperature: 6 cycles with 48ºC for 25 seconds, 6 cycles 52ºC for 20 seconds, and 20 cycles at 55ºC for 20 seconds, followed by a final extension step of 72ºC for 5 minutes. Cycle sequencing reactions were set up using PCR primers and the ABI Big Dye Terminator v1.1 kit and run on a ABI 3770 automated sequencer. The sequences were checked and assembled using Sequencher 4.1 (Gene code corporation), aligned with ClustalW and edited by eye in MacClade 4.0.

Phylogenetic analyses: comparing genes. Congruence between the *cox1* and *28S rDNA* trees was assessed by comparing support values for relationships in separate analyses. To judge conflict in the Bayesian analyses, we searched for nodes present in the 95% credible set of trees from one gene that were absent from the 95% credible set of trees from the other gene (4).

Morphometric analyses. Pictures were analyzed by geometric morphometrics, which uses the Cartesian coordinates of a set of topographically corresponding anatomical landmarks to compare forms (5). Using the TPS software (6), 6 landmarks were digitized on pictures of the trophi (Fig. 1). Landmarks were digitized only on one half to avoid redundancy in symmetric structures (7) and analyzed using Generalized Procrustes Analysis (GPA, (8)). Precision of landmark digitization and tangent space approximation were tested as in (7) and found to be satisfactory. The statistical significance of shape differences among populations and species of *Rotaria* was tested with a discriminant analysis on PCs explaining the first 95% of the variance, using jackknife cross-validation. Rotifer trophi do not change in size or shape with age (9, 10). We tested for significant differences in size among populations using an Analysis of Variance (ANOVA) and post-hoc tests (Games-Howell test, equal variance not assumed).

Clustering test for independent evolution. *Assumptions*. The signature of clusters is expected to take time to evolve after the onset of independent evolution and therefore we might fail to detect recent divergence and hence might underestimate the true number of entities. This might be less severe in asexuals than in sexuals, because any selective sweeps occurring in each separate species are expected to reduce population variation across the entire genome, and hence strengthen the pattern of clustering (11). However, if selective sweeps were rare and population sizes were large, expected coalescence times might be large and lead to low power to detect independently evolving entities with low to moderate sample sizes.

REFERENCES

1. Donner, J. (1965) *Rotifers* (Akademie-Verlag, Berlin).
2. Ricci, C. & Melone, G. (2000) *Hydrobiologia* **418,** 73-80.
3. Berzins, B. (1955) *Skrifter utgivna av Södra Sveriges Fiskeriförening Arsskrift* **1954-1955,** 107-243.
4. Buckley, T. R., Arensburger, P., Simon, C. & Chambers, G. K. (2002) *Syst. Biol.* **51,** 4-18.
5. Bookstein, F. L. (1991) *Morphometric Tools For Landmark Data: Geometry and Biology* (Cambridge Univ. Press, New York).
6. Rohlf, F. J. (2005) (Author, University of New York, Stony Brook, New York).
7. Fontaneto, D., Melone, G. & Cardini, A. (2004) *Ital. J. Zool.* **71,** 63-72.
8. Rohlf, F. J. & Slice, D. (1990) *Systematic Zoology* **39,** 40-59.
9. Fontaneto, D. & Melone, G. (2005) *Hydrobiologia* **546,** 213-221.
10. Fontaneto, D. & Melone, G. (2006) *Microscopy Research and Technique* **69,** 296-301.
11. Barraclough, T. G., Birky, C. W. & Burt, A. (2003) *Evolution* **57,** 2166-2172.
