# Supplementary material for: Independently Evolving Species in Asexual Bdelloid Rotifers
Source: PLoS Biol. 2007 Mar 20;5(4):e87. doi: 10.1371/journal.pbio.0050087 (PMC1828144; doi:10.1371/journal.pbio.0050087)
Supplement: Supplementary file 2 [file pbio.0050087.sd002.pdf]

PROTOCOL S2

*Tests for divergent selection*: *Rationale*. For a gene evolving neutrally within a set of isolated species, we expect the ratio of interspecific versus intraspecific changes in DNA sequence to be the same among all partitions (12, 13). In an asexual genome, sites experiencing purifying selection that is constant across species should display a relative excess of intraspecific changes versus interspecific changes, mainly on terminal branches, because purifying selection should be inefficient at purging mildly deleterious mutations (14). In contrast, any sites that have experienced divergent selection between species should display an excess of interspecific divergence relative to intraspecific divergence compared to a neutrally evolving partition (12, 13). We extend these predictions to morphological traits, assuming that trait variation is predominantly genetic. If rotifers have experienced divergent selection on trophi morphology between species, for example due to changes in habitat or resource use, we would expect a lower rate of evolution within species or clusters than between them relative to characters evolving neutrally. If instead morphology were under constant purifying selection but prone to a burden of mildly deleterious mutations, we might expect increased variation within species or clusters relative to neutral changes. Finally, if morphology were evolving neutrally, we would expect constant evolutionary rates across all branches of the tree.

*Assumptions and robustness*. 1) Error or randomly distributed phenotypic plasticity in trait values would tend to inflate intraspecific variation, i.e. to be assigned to the tips of the tree, reducing our ability to detect divergent selection, hence making the test conservative. Comparisons between lab-reared and wild-caught individuals (Table S6) rule out the possibility that species differences are solely due to plastic responses to environmental differences. 2) The test would have low power in asexuals if selective sweeps are very frequent, because the most recent sweep would have eradicated variation across all loci (13). Hence, this test assumes that sweeps are rare enough that neutral variation has had chance to re-accumulate in present populations. 3) Any biases affecting our sample, for example failing to sample a particular habitat with another genotype present, would affect which individuals are represented, not differences between DNA and morphology, therefore could not lead to artificial detection of divergent selection were none present among the sampled lineages. 4) A similar test could be devised for sexual populations, but this would need to consider the different ploidy levels of mitochondrial and nuclear genes and that variation in the morphological trait results from averaging several independent realizations of the coalescent

process. 5) Theory for sexual cases indicates that neutral expectations for quantitative traits are dependent only on the rate of additive mutations and relatively unaffected by other genetic details such as epistasis or dominance (15).

REFERENCES


12. McDonald, J. H. & Kreitman, M. (1991) *Nature* **351,** 652-654.
13. Nielsen, R. (2005) *Annual Review of Genetics* **39,** 197-218.
14. Paland, S. & Lynch, M. (2006) *Science* **311,** 990-992.
15. Lynch, M. & Hill, W. G. (1986) *Evolution* **40,** 915-935.
