# Supplementary material for: Independently Evolving Species in Asexual Bdelloid Rotifers
Source: PLoS Biol. 2007 Mar 20;5(4):e87. doi: 10.1371/journal.pbio.0050087 (PMC1828144; doi:10.1371/journal.pbio.0050087)
Supplement: Supplementary file 3 [file pbio.0050087.sd003.pdf]

SUPPORTING TEXT S1

Comparison of cox1 and 28S trees. Both genes yield strong support for monophyly of taxonomic species, but the relationships among species are poorly resolved (Figs. S1 to S3). One species, *R. neptunia*, was polyphyletic in the *28S rDNA* analysis (the two sequences grouping without strong support with different clades of *Rotaria rotatoria*) but monophyletic in the *cox1* mtDNA and combined analysis. *R. rotatoria* is split into several divergent lineages that are polyphyletic in the *cox1* and *28S* rDNA analyses but emerge as a single paraphyletic clade in the combined analysis, with the exception of one animal, R.rota.IT.8 which falls within the genus but not consistently with any other sub-lineage. While the status of this animal needs further investigation, the sequence was checked and found reliable hence we leave it in our analyses. The two species that live as epibionts of the water louse, *Asellus*, are recovered without strong support as sister species, nested within the paraphyletic *R. rotatoria*. Our three samples of *R. rotatoria* from the tropics are monophyletic. Other groupings were recovered with only moderate support and no bootstrap support in equivalent maximum parsimony analyses.

No trees within the 95% credible set of trees from the separate Bayesian analyses were compatible with the consensus tree or the most likely tree from the combined analysis (4). Sources of conflict included the basal relationships among species (in any case lacking support in the parsimony bootstrap analyses), the polyphyly of *R. neptunia* in the 28S analysis, and two cases of contradicted relationships within clusters (again lacking strong support in the more conservative parsimony bootstrap analysis). Although one possible source of conflict would be recombination between *cox1* and *28S* rDNA, without stronger support values conflict is most conservatively interpreted as a reconstruction artifact, for example non-independence among sites causing inflated support values. Evidence for a strong excess of deleterious changes to the *cox1* gene compared to levels observed in a comparable clade of facultatively sexual monogonont rotifers supports the asexuality of *Rotaria* and adds further weight to the wider evidence for bdelloids (Barraclough T.G. et al. in prep).

REFERENCES

4. Buckley, T. R., Arensburger, P., Simon, C. & Chambers, G. K. (2002) *Syst. Biol.* **51,** 4-18.
