# Supplementary material for: Independently Evolving Species in Asexual Bdelloid Rotifers
Source: PLoS Biol. 2007 Mar 20;5(4):e87. doi: 10.1371/journal.pbio.0050087 (PMC1828144; doi:10.1371/journal.pbio.0050087)
Supplement: Supplementary file 5 [file pbio.0050087.sg002.pdf]

Figure S2

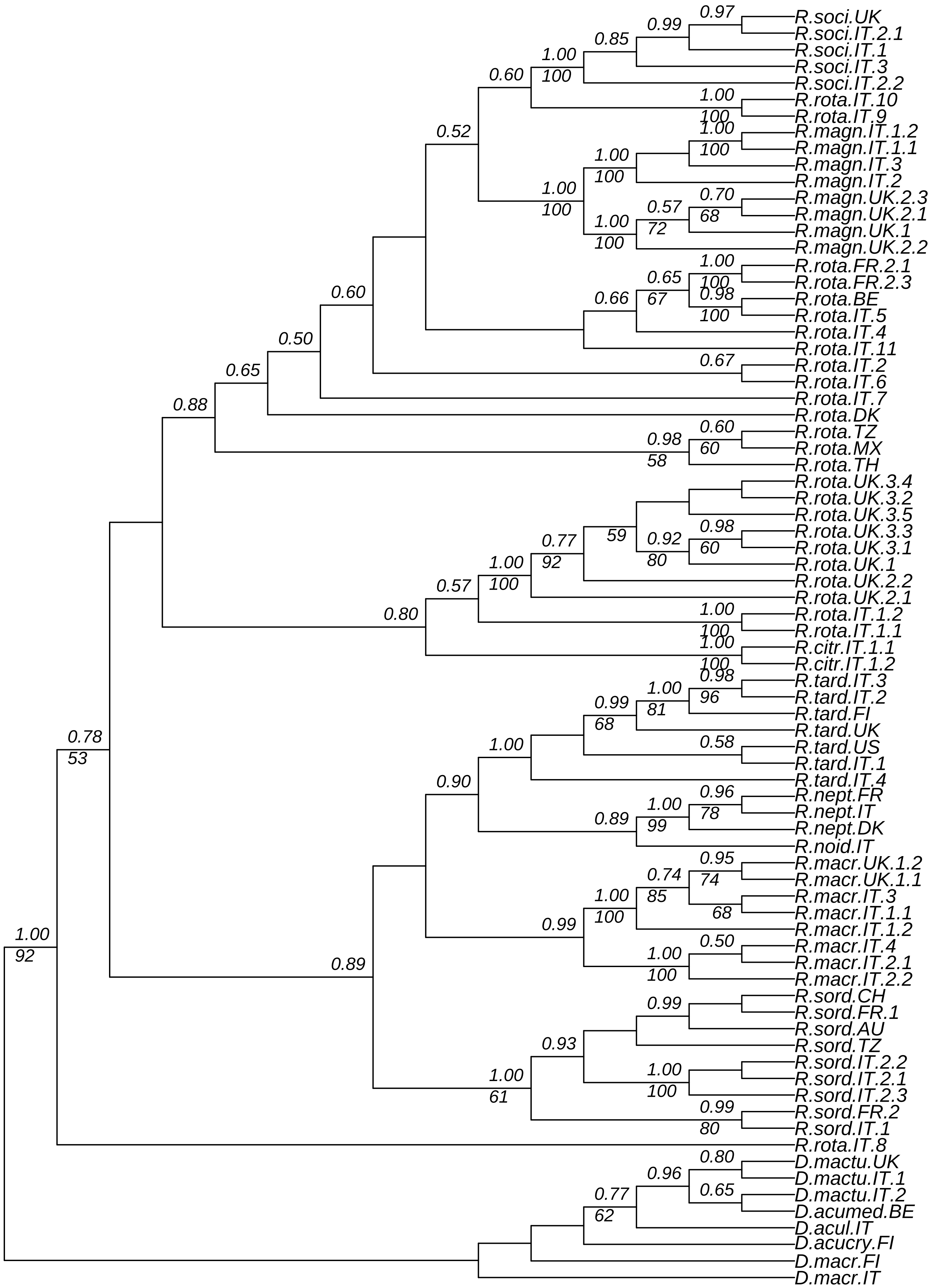

R.soci.UK
R.soci.IT.2.1
R.soci.IT.1
R.soci.IT.3
R.soci.IT.2.2
R.rota.IT.10
R.rota.IT.9
R.magn.IT.1.2
R.magn.IT.1.1
R.magn.IT.3
R.magn.IT.2
R.magn.UK.2.3
R.magn.UK.2.1
R.magn.UK.1
R.magn.UK.2.2
R.rota.FR.2.1
R.rota.FR.2.3
R.rota.BE
R.rota.IT.5
R.rota.IT.4
R.rota.IT.11
R.rota.IT.2
R.rota.IT.6
R.rota.IT.7
R.rota.DK
R.rota.TZ
R.rota.MX
R.rota.TH
R.rota.UK.3.4
R.rota.UK.3.2
R.rota.UK.3.5
R.rota.UK.3.3
R.rota.UK.3.1
R.rota.UK.1
R.rota.UK.2.2
R.rota.UK.2.1
R.rota.IT.1.2
R.rota.IT.1.1
R.citr.IT.1.1
R.citr.IT.1.2
R.tard.IT.3
R.tard.IT.2
R.tard.FI
R.tard.UK
R.tard.US
R.tard.IT.1
R.tard.IT.4
R.nept.FR
R.nept.IT
R.nept.DK
R.noid.IT
R.macr.UK.1.2
R.macr.UK.1.1
R.macr.IT.3
R.macr.IT.1.1
R.macr.IT.1.2
R.macr.IT.4
R.macr.IT.2.1
R.macr.IT.2.2
R.sord.CH
R.sord.FR.1
R.sord.AU
R.sord.TZ
R.sord.IT.2.2
R.sord.IT.2.1
R.sord.IT.2.3
R.sord.FR.2
R.sord.IT.1
R.rota.IT.8
D.mactu.UK
D.mactu.IT.1
D.mactu.IT.2
D.acumed.BE
D.acul.IT
D.acucry.FI
D.macr.FI
D.macr.IT
