# Supplementary material for: Independently Evolving Species in Asexual Bdelloid Rotifers
Source: PLoS Biol. 2007 Mar 20;5(4):e87. doi: 10.1371/journal.pbio.0050087 (PMC1828144; doi:10.1371/journal.pbio.0050087)
Supplement: Supplementary file 7 [file pbio.0050087.sg004.pdf]

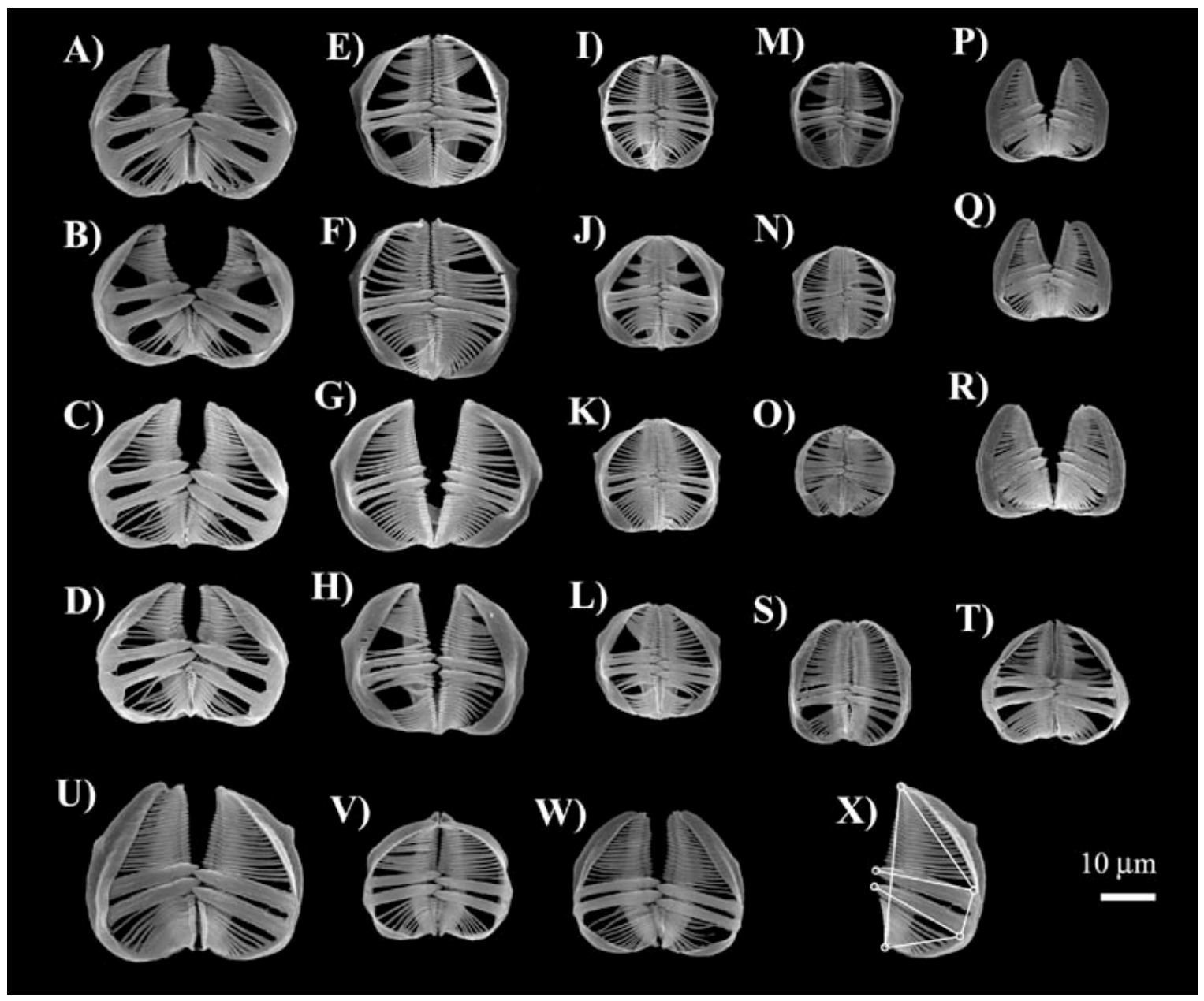
A)
E)
I)
M)
P)
B)
F)
J)
N)
Q)
C)
G)
K)
O)
R)
D)
H)
L)
S)
T)
U)
V)
W)
X)
10 μm
