# Supplementary material for: Independently Evolving Species in Asexual Bdelloid Rotifers
Source: PLoS Biol. 2007 Mar 20;5(4):e87. doi: 10.1371/journal.pbio.0050087 (PMC1828144; doi:10.1371/journal.pbio.0050087)
Supplement: Supplementary file 8 [file pbio.0050087.sg005.pdf]

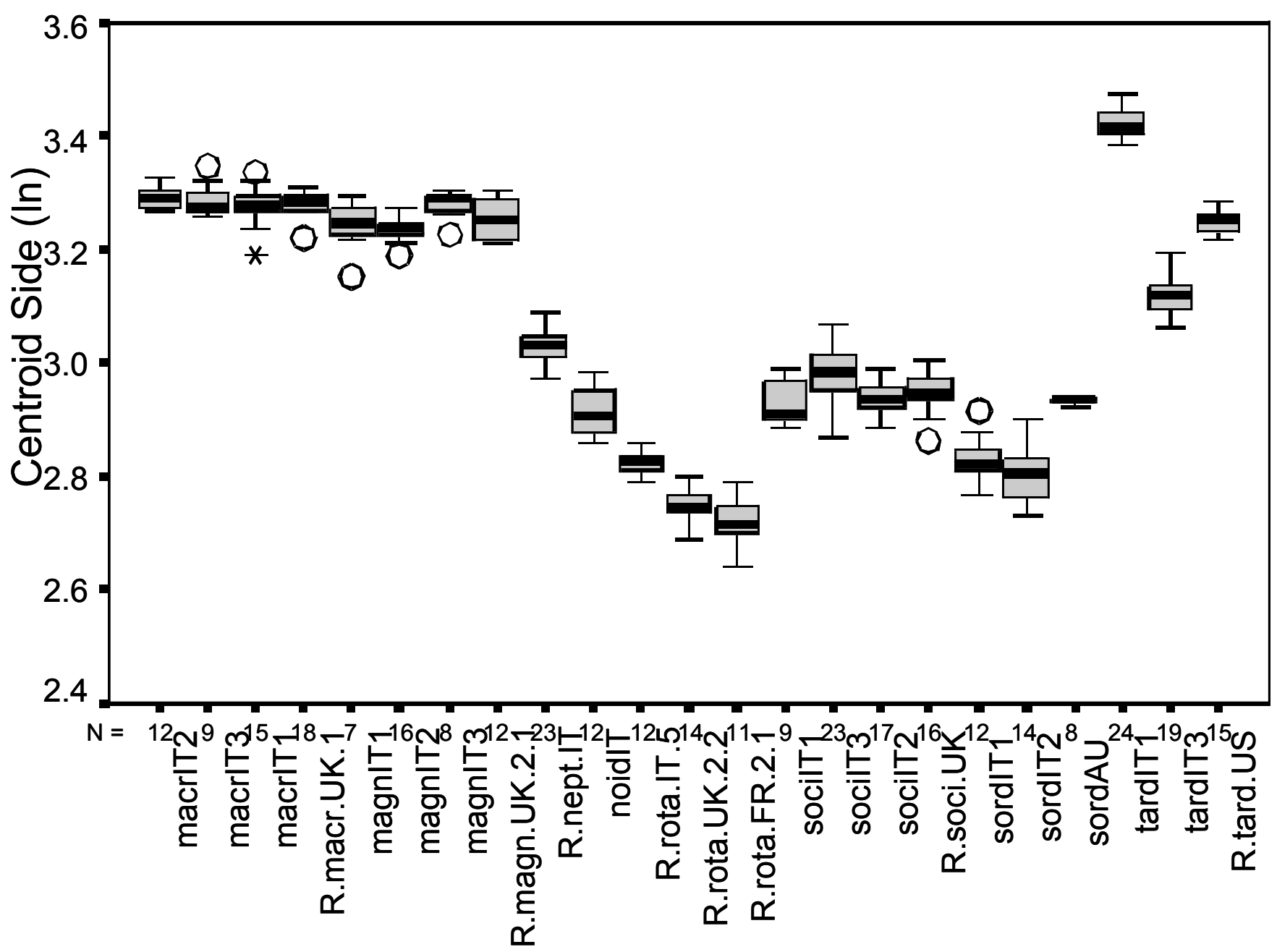
3.6
3.4
3.2
3.0
2.8
2.6
2.4
Centroid Side (ln)
N =
12
9
15
18
7
16
8
12
23
12
12
14
11
9
23
17
16
12
14
8
24
19
15
macrIT2
macrIT3
macrIT1
R.macr.UK.1
magnIT1
magnIT2
magnIT3
R.magn.UK.2.1
R.nept.IT
noidIT
R.rota.IT.5
R.rota.UK.2.2
R.rota.FR.2.1
sociIT1
sociIT3
sociIT2
R.soci.UK
sordIT1
sordIT2
sordAU
tardIT1
tardIT3
R.tard.US
