# Supplementary material for: Independently Evolving Species in Asexual Bdelloid Rotifers
Source: PLoS Biol. 2007 Mar 20;5(4):e87. doi: 10.1371/journal.pbio.0050087 (PMC1828144; doi:10.1371/journal.pbio.0050087)
Supplement: Supplementary file 9 [file pbio.0050087.st001.pdf]

TABLE S1. Locality records for DNA sequences and morphometric measurements. For detailed explanation of collection ID, see Fontaneto, D., Herniou, E., Barraclough, T. G. & Ricci, C. (2006) *Zoological Studies* In press.

a) DNA sequences and Genbank accession numbers.

Species	Sample name	Country	Locality	Date	Collection ID	Genebank code, cox1	Genebank code, 28S
D. aculeata (Ehrenberg, 1832)	D.acul.IT	Italy	Alagna VC	Jul 2004	A341	DQ656809	DQ656758
D. aculeata crystallina (Murray, 1908)	D.acucry.FI	Finland	Nuuksio National Park	Aug 2004	NUP1	DQ656808	DQ656757
D. aculeata medioaculeata (Janson, 1893)	D.acumed.BE	Belgium	Bruges	Nov 2003	BL2	DQ656810	DQ656759
D. macrostyla (Ehrenberg, 1838)	D.macr.FI	Finland	Nuuksio National Park	Aug 2004	NUP2	DQ656812	DQ656760
	D.macr.IT	Italy	Rassa VC	Jul 2004	A346	DQ656811	
D. macrostyla tuberculata (Gosse, 1886)	D.mactu.IT.1	Italy	Alagna VC	Jul 2004	A341	DQ656813	DQ656761
	D.mactu.IT.2	Italy	Campertogno VC	Oct 2004	ARG1	DQ656814	DQ656762
	D.mactu.UK	UK	New Forest, Hampshire	Oct 2004	NF5	DQ656815	DQ656763
R. citrina (Ehrenberg, 1838)	R.citr.IT.1.1	Italy	Novara NO	Jul 2005	A389	DQ656871	
	R.citr.IT.1.2	Italy	Novara NO	Jul 2005	A389	DQ656870	
R. macrura (Schrank, 1803)	R.macr.IT.1.1	Italy	Fontaneto NO	Aug 2004	RM1	DQ656816	DQ656777
	R.macr.IT.1.2	Italy	Fontaneto NO	Feb 2004	A327	DQ656818	
	R.macr.IT.2.1	Italy	Cavaglio NO	Aug 2004	RMAC	DQ656817	DQ656778
	R.macr.IT.2.2	Italy	Cavaglio NO	Feb 2004	A328	DQ656819	DQ656774
	R.macr.IT.3	Italy	Gattinara VC	Mar 2005	A371	DQ656881	
	R.macr.IT.4	Italy	San Bernardino CR	Nov 2005	A405	DQ656872	
	R.macr.UK.1.1	UK	New Forest, Hampshire	Oct 2004	NF8	DQ656820	DQ656776
	R.macr.UK.1.2	UK	New Forest, Hampshire	Oct 2004	NF8	DQ656821	DQ656775
R. magnacalcarata (Parsons, 1892)	R.magn.UK.1	UK	Kew Garden	Nov 2004	KG3	DQ656822	DQ656769
	R.magn.UK.2.1	UK	Silwood Park, Ascot	Sep 2004	S88	DQ656826	DQ656770
	R.magn.UK.2.2	UK	Silwood Park, Ascot	Sep 2004	S88	DQ656827	DQ656771
	R.magn.UK.2.3	UK	Silwood Park, Ascot	Sep 2004	S88	DQ656828	DQ656772
	R.magn.IT.1.1	Italy	Borromeo, Fontaneto NO	Jun 2004	A337	DQ656824	

	R.magn.IT.1.2	Italy	Borromeo, Fontaneto NO	Jun 2004	A337	DQ656825	DQ656767
	R.magn.IT.2	Italy	Serravalle Sesia VC	Aug 2004	A349	DQ656829	
	R.magn.IT.3	Italy	Fontanone, Fontaneto NO	Dec 2004	A362	DQ656823	DQ656768
R. neptunia (Ehrenberg, 1832)	R.nept.DK	Denmark	Aalborg, Jutland	Jul 2002	Genbank	AY218094	
	R.nept.FR	France	Vendee river, Pays de Loire	Sep 2003	V2	DQ656831	DQ656779
	R.nept.IT	Italy	Gorgonzola MI	Dec 2003	A313	DQ656830	DQ656773
R. neptunoida Harring, 1913	R.noid.IT	Italy	Rossa VC	May 2004	A324	DQ656856	
R. rotatoria (Pallas, 1766)	R.rota.BE	Belgium	Bruges	Nov 2003	BL2	DQ656832	DQ656784
	R.rota.DK	Denmark	Copenhagen	Apr 2001	Genbank	AY218093	
	R.rota.FR.1	France	Sully sur Loire	Jan 2004	CH9		DQ656756
	R.rota.FR.2.1	France	Languedoc - Roussillon	Sep 2004	SMV17	DQ656840	DQ656788
	R.rota.FR.2.2	France	Languedoc - Roussillon	Sep 2004	SMV17		DQ656805
	R.rota.FR.2.3	France	Languedoc - Roussillon	Sep 2004	SMV18	DQ656841	DQ656789
	R.rota.IT.1.1	Italy	Campertogno VC	Sep 2003	RR1	DQ656834	
	R.rota.IT.1.2	Italy	Campertogno VC	Oct 2003	A305	DQ656836	DQ656780
	R.rota.IT.2	Italy	Rassa VC	Jun 2004	A336	DQ656838	DQ656782
	R.rota.IT.3	Italy	Verbania VB	Oct 2004	IT2		DQ656764
	R.rota.IT.4	Italy	Pond, Fontaneto NO	Oct 2003	Rrot	DQ656835	
	R.rota.IT.5	Italy	Gorgonzola MI	Dec 2003	A313	DQ656837	DQ656781
	R.rota.IT.6	Italy	Cavaglio NO	Dec 2004	A363	DQ656833	DQ656783
	R.rota.IT.7	Italy	Sant'Agostino Lake, Varallo Sesia VC	May 2005	A382	DQ656876	
	R.rota.IT.8	Italy	Novara NO	Jul 2005	A389	DQ656877	
	R.rota.IT.9	Italy	Agogna river, Fontaneto NO	Feb 2005	A368	DQ656878	
	R.rota.IT.10	Italy	Marcurago di Arona NO	May 2005	A380	DQ656879	
	R.rota.IT.11	Italy	Verbania VB	Jul 2005	A387	DQ656880	
	R.rota.MX	Mexico	Alverado, Veracruz	Nov 2003	MX5	DQ656842	DQ656786
	R.rota.TZ	Tanzania	East Usambara	Sep 2003	TZ2	DQ656844	DQ656790
	R.rota.TH	Thailand	Koh Tan Island	Apr 2005	KS5	DQ656843	DQ656785
	R.rota.UK.1	UK	New Forest, Hampshire	Feb 2003	NF2	DQ656861	
	R.rota.UK.2.1	UK	New Forest, Hampshire	Oct 2004	NF5	DQ656839	DQ656787
	R.rota.UK.2.2	UK	New Forest, Hampshire	Oct 2004	NF5	DQ656862	
	R.rota.UK.2.3	UK	New Forest, Hampshire	Oct 2004	NF5		DQ656766
	R.rota.UK.3.1	UK	Wisley, Surrey	Mar 2003	W4	DQ656863	DQ656795
	R.rota.UK.3.2	UK	Wisley, Surrey	Mar 2003	W4	DQ656864	

	R.rota.UK.3.3	UK	Wisley, Surrey	Mar 2003	W4	DQ656865	DQ656796
	R.rota.UK.3.4	UK	Wisley, Surrey	Mar 2003	W4	DQ656866	DQ656797
	R.rota.UK.3.5	UK	Wisley, Surrey	Mar 2003	W4	DQ656867	
	R.rota.UK.3.6	UK	Wisley, Surrey	Mar 2003	W4		DQ656807
R. socialis (Kellicott, 1888)	R.soci.IT.1	Italy	Borromeo, Fontaneto NO	Jun 2004	A337	DQ656845	DQ656798
	R.soci.IT.2.1	Italy	Fontanone, Fontaneto NO	Aug 2004	A344	DQ656846	DQ656799
	R.soci.IT.2.2	Italy	Fontanone, Fontaneto NO	Dec 2004	A362	DQ656849	
	R.soci.IT.3	Italy	Serravalle Sesia VC	Aug 2004	A349	DQ656848	
	R.soci.UK	UK	Silwood Park, Ascot	Oct 2004	S88	DQ656847	DQ656800
R. sordida (Western, 1893)	R.sord.AU	Australia	Lamington	Aug 2004	OZ4	DQ656853	DQ656794
	R.sord.CH	Switzerland	Neuchatel	Sep 2003	SW11	DQ656854	
	R.sord.FR.1	France	les Sables d'Olonnes, Pays de Loire	Aug 2003	P10	DQ656850	DQ656793
	R.sord.FR.2	France	Languedoc - Roussillon	Feb 2004	SMV13	DQ656855	
	R.sord.IT.1	Italy	Borromeo, Fontaneto NO	Jun 2004	A338	DQ656852	DQ656792
	R.sord.IT.2.1	Italy	Sant'Agostino Lake, Varallo Sesia VC	May 2005	A384	DQ656873	
	R.sord.IT.2.2	Italy	Sant'Agostino Lake, Varallo Sesia VC	May 2005	A384	DQ656874	
	R.sord.IT.2.3	Italy	Sant'Agostino Lake, Varallo Sesia VC	May 2005	A384	DQ656875	
	R.sord.SW	Sweden	Stockholm	Oct 2004	ST1		DQ656806
	R.sord.TZ	Tanzania	East Usambara	Sep 2003	A314	DQ656851	DQ656791
	R.sord.UK	UK	New Forest, Hampshire	Oct 2004	NF15		DQ656765
R. tardigrada (Ehrenberg, 1832)	R.tard.FI	Finland	Nuuksio National Park	Aug 2004	NUP2	DQ656860	DQ656803
	R.tard.IT.1	Italy	Piode VC	Aug 2004	A348	DQ656857	DQ656801
	R.tard.IT.2	Italy	Verbania VB	Oct 2004	IT1	DQ656858	DQ656802
	R.tard.IT.3	Italy	Mercurago di Arona NO	May 2005	A376	DQ656869	
	R.tard.IT.4	Italy	Sant'Agostino Lake, Varallo Sesia VC	May 2005	A382	DQ656868	
	R.tard.UK	UK	New Forest, Hampshire	Oct 2004	NF5	DQ656859	
	R.tard.US	USA	Jamaica plain, Boston MA	Nov 2004	BO1	DQ656882	DQ656804

b) SEM and morphometric measurements. Individuals were measured from population samples from the field (P) or clonal samples derived from a single wild individual (C). Population sample names match those for DNA sequences derived from the same population, e.g. macrIT1 is a sample from the same population as the sequence R.macr.IT.1.

species	Sample name	locality	date	# SEM measured	C/P
R. macrura (Schrank, 1803)	macrIT1	Italy	Dec 2003	15	P
	macrIT2	Italy	Aug 2004	12	P
	macrIT3	Italy	Mar 2005	9	P
	R.macr.UK.1	UK	Oct 2004	18	C
R. magnacalcarata (Parsons, 1892)	magnIT1	Italy	Jun 2004	7	P
	magnIT2	Italy	Aug 2004	16	P
	magnIT3	Italy	Dec 2004	8	P
	R.magn.UK.2.1	UK	Oct 2004	12	C
R. neptunia (Ehrenberg, 1832)	R.nept.IT	Italy	Dec 2003	23	C
R. neptunoida Harring, 1913	noidIT	Italy	May 2004	12	P
R. rotatoria (Pallas, 1766)	R.rota.FR.2.1	France	Sep 2004	11	C
	R.rota.IT.5	Italy	Dec 2003	12	C
	R.rota.UK.2.2	UK	Oct 2004	14	C
R. socialis (Kellicott, 1888)	sociIT1	Italy	Jun 2004	9	P
	sociIT2	Italy	Aug 2004	17	P
	sociIT3	Italy	Aug 2004	23	P
	R.soci.UK	UK	Oct 2004	16	C
R. sordida (Western, 1893)	sordAU	Australia	Aug 2004	8	P
	sordIT1	Italy	Jun 2004	12	P
	sordIT2	Italy	May 2005	14	P
R. tardigrada (Ehrenberg, 1832)	tardIT1	Italy	Aug 2004	24	P
	tardIT3	Italy	May 2005	19	P
	R.tard.US	USA	Nov 2004	15	C
