# Supplementary material for: Independently Evolving Species in Asexual Bdelloid Rotifers
Source: PLoS Biol. 2007 Mar 20;5(4):e87. doi: 10.1371/journal.pbio.0050087 (PMC1828144; doi:10.1371/journal.pbio.0050087)
Supplement: Supplementary file 10 [file pbio.0050087.st002.pdf]

TABLE S2. Traditional characteristics used to distinguish *Rotaria* species. Data from Donner, J. (1965) *Rotifers* (Akademie-Verlag, Berlin) and Ricci, C. (1998) *Hydrobiologia* **388,** 321-326.

Species	body length (micron)	foot	spurs	toes	rostrum	eyes	detritus on trunk	wet habitat	dry habitat	benthos	plankton	epibiont	running water	lakes and ponds	anhydrobiotic capabilities
citrina	600-1000	short	short	short	short	present	no	x		x				x	unknown, but plausibly poor
macrura	600-800	long	short	short	long	present	no	x		x	x			x	unknown, but plausibly poor
magnacalcarata	600-800	long	long	short	short	absent	no	x		x		x		x	unknown, but plausibly poor
neptunia	700-1600	very long	short	long	short	present	no	x		x				x	bad
neptunoida	400-1000	long	short	long	long	present	no	x		x				x	quite good in embryos
rotatoria	230-1090	medium	short	short	short	present	no	x		x	x		x	x	bad
socialis	350-650	long	medium	short	short	absent	no	x		x		x		x	unknown, but plausibly poor
sordida	340-650	short	long	short	long	absent	yes	x	x	x				x	good
tardigrada	363-700	short	long	long	long	present	yes	x		x				x	good
