# Supplementary material for: Independently Evolving Species in Asexual Bdelloid Rotifers
Source: PLoS Biol. 2007 Mar 20;5(4):e87. doi: 10.1371/journal.pbio.0050087 (PMC1828144; doi:10.1371/journal.pbio.0050087)
Supplement: Supplementary file 11 [file pbio.0050087.st003.pdf]

TABLE S3. Discriminant analysis of trophi shape. The analysis is based on the first five PCs (total variance explained: PC1, 51.75%; PC2, 19.91%; PC3, 12.31%; PC4, 8.18%; PC5, 4.94%). Cell entries show the percentage of animals from the population in column heading classified as belong to the population given by the row name. Specimens correctly classified at population level fall along the diagonal; specimens correctly classified at species level fall within the rectangular boxes; all other specimens are misclassified by the analysis. For example 58% of macrIT2 individuals were correctly classified to population, whereas 25% were classified as belonging to macrIT1.

	macrIT2	macrIT3	macrIT1	R.macr.UK.1	magnIT1	magnIT2	magnIT3	R.magn.UK.2.1	R.nept.IT	noidIT	R.rota.IT.5	R.rota.UK.2.2	R.rota.FR.2.1	sociIT1	sociIT3	sociIT2	R.soci.UK	sordIT1	sordIT2	sordAU	tardIT1	tardIT3	R.tard.US
macrIT2	58	0	25	8	0	0	0	0	0	0	0	0	0	0	0	0	0	0	0	0	0	8	0
macrIT3	0	67	33	0	0	0	0	0	0	0	0	0	0	0	0	0	0	0	0	0	0	0	0
macrIT1	7	13	67	13	0	0	0	0	0	0	0	0	0	0	0	0	0	0	0	0	0	0	0
R.macr.UK.1	0	11	17	72	0	0	0	0	0	0	0	0	0	0	0	0	0	0	0	0	0	0	0
magnIT1	0	0	0	0	43	14	0	14	0	0	0	0	0	14	0	0	14	0	0	0	0	0	0
magnIT2	0	0	0	0	6	19	13	19	0	0	6	0	6	0	6	19	6	0	0	0	0	0	0
magnIT3	0	0	0	0	0	0	63	13	0	0	0	0	0	13	13	0	0	0	0	0	0	0	0
R.magn.UK.2.1	0	0	0	0	0	8	8	42	0	0	8	0	0	0	17	17	0	0	0	0	0	0	0
R.nept.IT	0	0	0	0	0	0	0	0	91	0	0	0	0	0	0	0	0	4	4	0	0	0	0
noidIT	0	0	0	0	0	0	0	0	0	100	0	0	0	0	0	0	0	0	0	0	0	0	0
R.rota.IT.5	0	0	0	0	0	8	8	0	0	0	58	0	8	8	0	8	0	0	0	0	0	0	0
R.rota.UK.2.2	0	0	0	0	0	0	0	0	0	0	0	100	0	0	0	0	0	0	0	0	0	0	0
R.rota.FR.2.1	0	0	0	0	0	0	0	0	0	0	9	0	18	9	18	9	27	9	0	0	0	0	0
sociIT1	0	0	0	0	0	0	0	0	0	0	11	0	11	44	22	0	11	0	0	0	0	0	0
sociIT3	0	0	0	0	13	4	0	13	0	0	4	4	9	4	26	4	9	0	9	0	0	0	0
sociIT2	0	0	0	0	6	0	0	6	0	0	0	6	0	0	24	53	6	0	0	0	0	0	0
R.soci.UK	0	0	0	0	6	0	0	13	0	0	0	0	25	0	6	13	38	0	0	0	0	0	0
sordIT1	0	0	0	0	0	0	0	0	0	0	0	0	0	0	0	0	0	67	25	8	0	0	0
sordIT2	0	0	0	0	0	0	0	0	0	0	0	0	0	0	0	0	0	29	64	0	7	0	0
sordAU	0	0	0	0	0	0	0	0	0	0	0	0	0	0	0	0	0	0	0	100	0	0	0
tardIT1	0	0	0	0	0	0	0	0	0	0	0	0	0	0	0	0	0	0	4	0	63	13	21
tardIT3	0	0	0	0	0	0	0	0	0	0	0	0	0	0	0	0	0	0	0	0	5	90	5
R.tard.US	0	0	0	0	0	0	0	0	0	0	0	0	0	0	0	0	0	0	0	0	13	13	73
