# Supplementary material for: Independently Evolving Species in Asexual Bdelloid Rotifers
Source: PLoS Biol. 2007 Mar 20;5(4):e87. doi: 10.1371/journal.pbio.0050087 (PMC1828144; doi:10.1371/journal.pbio.0050087)
Supplement: Supplementary file 12 [file pbio.0050087.st004.pdf]

TABLE S4. Comparison of models of single versus multiple independently evolving entities within *Rotaria*. Analyses were repeated on tree obtained from the combined and separate gene partitions in turn. The independent evolution model included 5 free parameters: the threshold genetic distance, *T*, from the present defining the transition from between entity to within entity branching, and separate branching rates, λ_1 and λ_2, and scaling parameters, p_1 and p_2, for branching between and within entities respectively. The null model includes 2 free parameters. The number of inferred independently evolving entities represented in the sample, and the number of those that are represented by single individuals, are shown with confidence limits within 2 log likelihoods of the maximum presented in brackets. The cluster provides a significantly better fit to the data for the combined and cox1 trees in log likelihood ratio tests with three degrees of freedom, * indicates $p<0.05$, *** indicates $p<0.0001$

Tree	Model	Log likelihood	T Subs/site	λ_1	λ_2	p_1	p_2	# of independent entities	# of entities with > 1 individual sampled
Combined	Null	311.6		3.02		0.60		1	0
	Clusters***	327.0	0.045	10.3	108.8	0.64	0.00	43 (41-45)	13 (13-13)
cox1	Null	257.8		3.04		0.56		1	0
	Clusters***	282.4	0.041	16.7	106.2	0.37	0.00	38 (37-40)	13 (13-13)
28S rDNA	Null	162.3		7.9		0.45		1	0
	Clusters	162.7	0.144	0.14	9.9	7.9	0.41	2 (1-21)	2 (1-8)
