# Supplementary material for: Independently Evolving Species in Asexual Bdelloid Rotifers
Source: PLoS Biol. 2007 Mar 20;5(4):e87. doi: 10.1371/journal.pbio.0050087 (PMC1828144; doi:10.1371/journal.pbio.0050087)
Supplement: Supplementary file 13 [file pbio.0050087.st005.pdf]

TABLE S5. Evolutionary rates of changes in trophi size and shape within and between clusters and species. Rates were reconstructed on the combined analysis tree topology with branch lengths optimized as synonymous substitutions per codon. Results using other combinations of topology and branch lengths are qualitatively the same. Model 1 represents a single rate across tree (β_1), model 2 represents a different rate within taxonomic species (β_1) and between them (β_2), model 3 represents a different rate within clusters (β_1) and between them (β_2), and model 4 represents different rates within clusters (β_1), between clusters within a taxonomic species (β_2) and between taxonomic species (β_3). Units are variance in trait per unit branch length. Confidence limits on parameter estimates from solutions within 2 log likelihood units of the maximum likelihood solution are shown. The preferred model for each trait is indicated by asterisks, with * indicating $p<0.05$, ** for $p<0.01$, *** for $p<0.001$ compared to the most likely nested model in log likelihood ratio tests.

Trait	Model	Log likelihood	β_1	β_2	β_3
Size	1	75.2	0.012±0.004		
	2 **	78.7	0.006±0.003	0.031±0.015	
	3	78.4	0.0044±0.0014	0.016±0.0061	
	4	79.1	0.0044±0.0018	0.0071±0.0025	0.029±0.010
PC1	1	106.2	0.0030±0.0011		
	2	108.7	0.0054±0.0023	0.00074±0.00023	
	3	110.4	0.0058±0.0028	0.0016±0.0003	
	4 ***	123.4	0.0058±0.0022	0.00008±0.00003	0.0043±0.0009
PC2	1	140.6	0.0006±0.0002		
	2	143.1	0.00038±0.00014	0.0011±0.0002	
	3	140.6	0.00058±0.0003	0.00063±0.00012	
	4 *	145.7	0.00059±0.00026	0.00016±0.00007	0.0011±0.0003
