# Supplementary material for: Independently Evolving Species in Asexual Bdelloid Rotifers
Source: PLoS Biol. 2007 Mar 20;5(4):e87. doi: 10.1371/journal.pbio.0050087 (PMC1828144; doi:10.1371/journal.pbio.0050087)
Supplement: Supplementary file 14 [file pbio.0050087.st006.pdf]

TABLE S6. The effects of sampling type (clonal versus population sample) on the mean and variance of size and shape of trophi within each sample.

a) Nested analysis of variance with sampling type as fixed effect and species as random effect. Sampling type has non-significant effect on the mean value of the response variable in each case. There are 326 observations and 8 species groups in each case.

	terms	coefficient	t	p
Centroid size	intercept	3.1	41.3	0.00
	sampling type	-0.012	-1.13	0.26
PC1	intercept	-0.017	-0.59	0.56
	sampling type	0.0044	1.34	0.18
PC2	intercept	0.0030	0.18	0.86
	sampling type	-0.0091	-1.43	0.15

b) Comparisons of analyses of variance with species as a random effect and either including or excluding sampling type as a nested random effect within species. In all cases, the more complex model is not a significantly better fit for the data, showing that sampling type has no significant effect on the variance in each response variable.

Response	N	d.f.	Log likelihood	Log L ratio	p
Centroid size	Species	3	384.9	<0.00	1.0
	Species/type	4	384.9		
PC1	Species	3	759.62	<0.00	1.0
	Species/type	4	759.62		
PC2	Species	3	549.9	1.15	0.28
	Species/type	4	550.46		

Analyses conducted in the R statistical programming language, using the "lme" function.
